# Acid erosion, surface topography, and fluoride release of glass ionomer cements subjected to erosive challenge

**DOI:** 10.1590/0103-644020266840

**Published:** 2026-07-24

**Authors:** Fernanda Oliveira Miranda Tavares, Carolina Mara Geraldino Monteiro, Felipe Marchiori Guimarães, Renan Rocha da Silva, Andrea Vaz Braga Pintor, Lucianne Cople Maia

**Affiliations:** 1 Universidade Federal do Rio de Janeiro, Faculdade de Odontologia, Departamento de Odontopediatria e Ortodontia, Rio de Janeiro, Rio de Janeiro, Brasil.

**Keywords:** Glass ionomer cement, Acid erosion, Dental materials, Fluorine

## Abstract

To evaluate and compare the acid erosion resistance, surface topography, and fluoride release of restorative glass ionomer cements (GICs) subjected to erosive challenge. Specimens (n=6) of Vidrion R® (VD), Maxxion R® (MA), Vitro Molar® (VM), and GlasIonomer FX ULTRA® (GI) were prepared and stored in water at 5°C/24h. Baseline measurements of thickness, weight, and surface roughness (linear - Ra; volumetric - Sa) were obtained using a non-contact 3D profilometer. Specimens were immersed in 30 mL of a lactic acid buffer (pH 2.74) at 37°C/24h, after which final thickness (eroded depth), mass (eroded mass), and surface roughness were measured. Fluoride release was evaluated in duplicate using a calibrated fluoride-specific electrode. Data were analyzed using Jamovi 2.2.5 (p<0.05). All GICs showed a reduction in thickness; however, only VM exhibited an eroded depth below 0.17 mm (0.10 ± 0.03 mm, per ISO standards). Regarding mass loss, VD experienced the highest reduction and VM the lowest (p<0.05), with GI and MA showing similar losses (p>0.05). Regarding surface roughness, VD showed a reduction in Ra, whereas the other GICs showed increases (p>0.05). All specimens demonstrated a significant increase in Sa, with MA showing the greatest change (p<0.05). In fluoride release, MA and VD had the highest values (98.6 ± 11.8 and 91.4 ± 8.16), GI was intermediate (66.1 ± 4.12), and VM had the lowest (38.4 ± 9.97) (p<0.05). Although all products released fluoride and underwent significant changes in thickness, mass, and surface roughness under erosive challenge, only VM demonstrated adequate acid erosion resistance according to ISO standards**.**

## Resumo

Avaliar e comparar a resistência à erosão ácida, topografia de superfície e liberação de fluoreto de cimentos de ionômero de vidro (CIV) restauradores submetidos a desafio erosivo. Espécimes (n=6) de Vidrion R® (VD), Maxxion R® (MA), Vitro Molar® (VM), e GlasIonomer FX ULTRA® (GI) foram preparados e estocados em água a 5°C por 24h. Valores iniciais de espessura, massa e rugosidade de superfície (linear - Ra; volumétrica - Sa) foram obtidos usando perfilômetro 3D de não-contato. Os espécimes foram imersos em 30 mL de solução de ácido láctico (pH 2.74), a 37°C por 24h, após o que espessura (profundidade erodida), massa (massa erodida) e topografia de superfície (Ra e Sa) finais foram medidas. A liberação de fluoreto foi avaliada em duplicada utilizando um eletrodo flúor-específico calibrado. Os dados foram analisados utilizando Jamovi 2.2.5 (p<0,05). Todos os CIVs mostraram uma redução na espessura; porém, apenas VM exibiu uma profundidade de erosão abaixo de 0,17 mm (0,10 ± 0,03 mm, padrão ISO). Relativo à perda de massa, VD obteve a maior redução e VM a menor (p>0,05). Em termos da rugosidade de superfície, VD mostrou redução da Ra, enquanto os outros CIVs exibiram aumento (p>0,05). Todos os espécimes demonstraram aumento significativo da Sa, com MA mostrando a maior mudança (p<0,05). Na liberação de fluoreto, MA e VD tiveram os maiores valores (98,6 ± 11,8 and 91,4 ± 8,16), GI foi intermediário (66,1 ± 4,12) e VM o menor (38,4 ± 9,97) (p<0,05). Embora todos os produtos tenham liberado fluoreto e sofrido alterações significativas na espessura, massa e rugosidade da superfície sob desafio erosivo, somente o VM demonstrou resistência adequada à erosão ácida de acordo com os padrões ISO.

**Figure:**
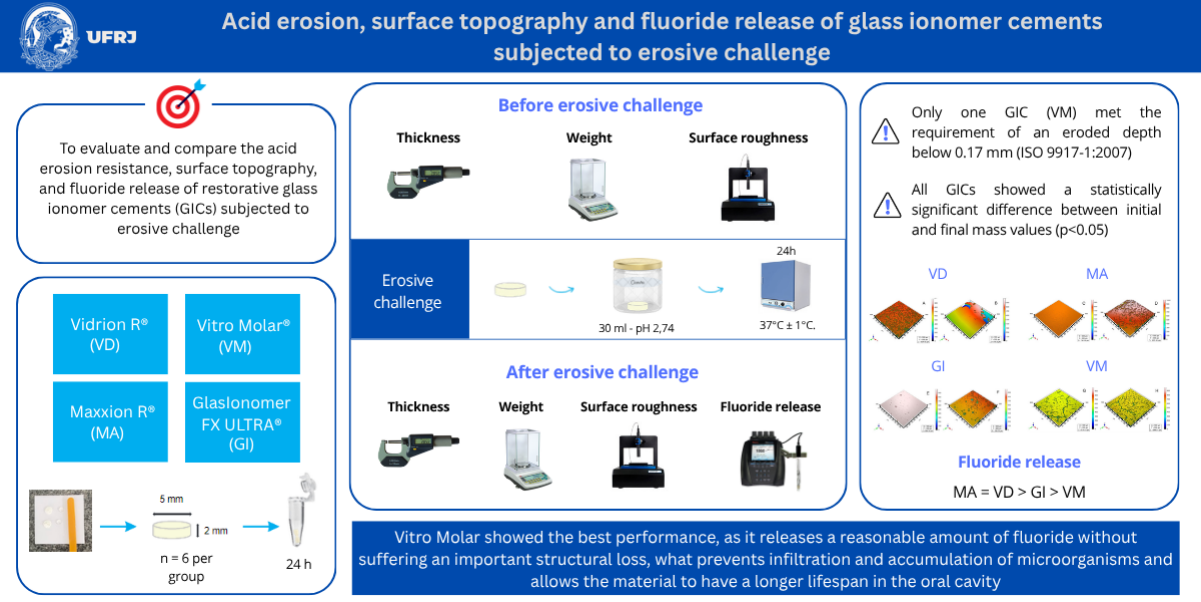

## Introduction

Glass ionomer cement (GIC) was developed by Wilson and Kent and has been marketed since the 1970s.^1^ It is a versatile restorative material, widely used in Dentistry, especially in Pediatric Dentistry.^2^ GICs are composed of powder and liquid that, when mixed in the correct proportions, initiate an acid-base reaction.^3^ ^)^ The powder consists of silica (SiO₂), alumina (Al₂O₃), calcium fluoride (CaF₂), sodium fluoride (NaF), aluminum fluoride (AlF₃), calcium phosphate (Ca₃(PO₄)₂), or aluminum phosphate (AlPO₄). The liquid, in turn, is composed of polyacrylic acid and copolymers of itaconic, maleic, or tricarboxylic acids.^4^

Among the characteristics that make it an attractive material are the release of fluoride in the oral environment, chemical adhesion to enamel and dentin, ease of handling, a thermal expansion coefficient similar to that of dental tissue, and biocompatibility.^5^ ^,^ ^6^ These advantages make it suitable for use in Atraumatic Restorative Treatment (ART), performing repairs of the crown margin and restorations in enamel and dentin, filling cores, sealing pits and fissures, as well as lining and bases for cavities that will be restored.^7^ ^)^ However, glass ionomer cement is sensitive to syneresis and imbibition, is less resistant to tensile stress and wear compared to other restorative materials, and is more opaque and less glossy than composite resin.^4^ ^,^ ^8^ Due to these characteristics, its use is not recommended in restorations involving marginal ridges, pin-retained cores, cusp replacement, and incisal restorations.^7^ ^,^ ^9^

There is a wide variety of GICs available on the market, with different powder/liquid ratios, compositions, and viscosities.^10^ Studies comparing the properties of various commercial brands aim to highlight and assist in the identification of the most efficacious materials and serve as a basis for future studies.^11^ ^,^ ^12^ ^,^ ^13^

The oral environment is subject to acidic conditions, whether from extrinsic acids, such as the consumption of acidic foods, or intrinsic sources, such as gastroesophageal reflux and byproducts of polysaccharide metabolism by microorganisms present in the oral microbiota, such as lactic acid, which contribute to the demineralization of dental structures.^14^ ^,^ ^15^ Exposure to acids can affect the surface of dental materials used in restorations, increasing surface roughness and leading to biofilm accumulation, gingival inflammation, and restoration staining.^15^ ^,^ ^16^

Due to its fluoride release capacity, which produces a biological response in the adjacent tissues, glass ionomer cement is considered a bioactive material.^12^ The released fluoride has clinical significance, as it helps reduce the demineralization process of dental substrates, and its release increases in acidic pH^.(^ ^12^ ^,^ ^17^

In the present study, three national restorative glass ionomer cements were chosen, as they are widely used in both public and private services in Brazil, and it is therefore important to determine whether they constitute adequate options for clinical use. As an imported product, GlasIonomer FX ULTRA was chosen, as there are no studies in the scientific literature on the latter. Thus, the aim of this study was to evaluate and compare the resistance to acid erosion, surface topography, and fluoride release of restorative glass ionomer cement (GICs) subjected to erosive challenge. The null hypothesis to be tested is that there are no differences among the studied properties and variables regarding the evaluated restorative materials.

## Materials and methods

### Study design

The present laboratory experimental study, conducted at the *XXX*, compared national restorative glass ionomer cements (GIC) Vidrion R® (VD), Maxxion R® (MA), and Vitro Molar® (VM) with the imported GIC GlasIonomer FX ULTRA® (GI) (Table 1) regarding acid erosion, surface topography, and fluoride release under erosive challenge.

**Table 1:** Specifications of the glass ionomer cements used in the study.

Material	Manufacturer (batch number)	Composition	Powder/liquid ratio
Vidrion R (VD)	SSWhite - Rio de Janeiro/RJ - Brazil (111221)	Powder: Sodium fluosilicate, calcium, aluminum, barium sulfate, polyacrylic acid, pigments; Liquid: Tartaric acid, water	5.8:1
Maxxion R (MA)	FGM - Joinvile/SC - Brazil (61021)	Powder: Fluoro-aluminosilicate glass; Liquid: Polyacrylic acid, calcium fluoride, water	1.5:1
Vitro Molar (VM)	DFL - Rio de Janeiro/RJ - Brazil (21060632)	Powder: Fluorosilicate, barium and aluminum, dehydrated polyacrylic acid, and iron oxide; Liquid: Polyacrylic acid, tartaric acid, distilled water	2.9:1
GlasIonomer FX ULTRA (GI)	Shofu Inc. - Kyoto - Japan (012001)	Powder: Fluoro-aluminosilicate glass and pigments; Liquid: Polyacrylic acid, tricarboxylic acid, tartaric acid, and copolymer	2.7:1

### Acid erosion test (erosive challenge)

### Preparation of the erosive solution

The erosive solution, with a pH of 2.74, was prepared following the Standard Operating Procedure (SOP) of “Laboratório Multidisciplinar de Pesquisa em Odontologia da Faculdade de Odontologia” *da UFRJ (LAMPO/FO-UFRJ)* for the acid erosion test^12^, using lactic acid and lactate, which were previously calculated and dissolved in water. After preparation, the solution was stored in a refrigerator, protected from light, until use.

### Preparation of the specimens and erosive challenge

According to ISO standards, for studies assessing the erosive challenge of GICs, the sample size is 5 specimens per group (ISO 9917:2017). To prevent bias in statistical analyses due to sample loss, the authors decided to work with 6 specimens per group. The specimens (n=6 per group) were prepared by a single trained operator, following the manufacturer's recommendations, and placement into pre-fabricated silicone molds with disc-shaped holes of 2 mm in height and 5 mm in diameter, in accordance with ISO 9917:2017. The molds were kept on a flat surface, the holes were filled with GIC with the aid of a dental spatula type 1 to prevent forming air bubbles, respecting the manipulation and working times, and covered with a mylar strip and a glass plate to ensure a smooth and uniform surface and to control the interference of humidity and porosity in the setting mechanism of the specimen. After the manufacturers' indicated setting time, the specimens were individually stored in Eppendorf tubes containing reverse osmosis water for 24 hours.

Next, the specimens underwent initial evaluation of thickness, mass, and surface topography. Subsequently, each specimen was immersed in 30 mL of the erosive solution for 24 hours, at 37°C ± 1°C. After the immersion time, each specimen was removed, washed with reverse osmosis water (ISO 3696:1987), and subjected to the same evaluations performed before the erosive challenge.

### Measurement and calculation of initial thickness (d_0_), final thickness (d_t_), and eroded depth (d)

The thickness of each specimen was measured at five points using a digital micrometer (SYNTEK, Guangdong, China) by a single trained operator. The averages and standard deviations of the five measurements for each specimen and the total of the specimens were calculated. The measurement was performed before (*D*_*0*_ ) and after the erosive challenge (*D*_*T*_ ). The eroded depth (*D*) was evaluated and expressed in millimeters using the equation *D = D*_*T*_ *- D*_*0*_ .

### Measurement and calculation of initial mass (m_0_), final mass (m_t_), and eroded mass (m)

The mass of each specimen was obtained using a precision scale with four digits (AD500, MARTE, Santa Rita do Sapucaí, Minas Gerais, Brazil), by a single trained operator, before (*M*_*0*_ ) and after the erosive challenge (*M*_*T*_ ). The eroded mass (*M*) was evaluated and expressed in grams using the equation *M = M*_*T*_ *- M*_*0*_ .

### Surface topography evaluation

The surface topography of the specimens was obtained using non-contact three-dimensional optical profilometry (Nanovea PS50 Optical, NANOVEA Inc., Irvine, California, USA), in which the linear roughness (Ra) and volumetric roughness (Sa) of each specimen were evaluated before and after the erosive challenge. The acquisition scans were performed with a chromatic confocal sensor using an axial white-light source, at a scanning speed of 3 µ/s and a refractive index of 10,000. For each specimen, an area of 1 mm x 1 mm was measured at the center of the sample. To estimate Ra (ISO 4287), three horizontal linear measurements were taken in the area of interest, and the average was used to determine *Ra*_*0*_ and *Ra*_*T*_ . The difference in linear roughness for all groups was calculated as: *Ra = Ra*_*T*_ *- Ra*_*0*_ . For the evaluation of Sa (ISO 25178), a measurement was taken in the area of interest for each specimen. The difference in volumetric roughness for all groups was calculated as: *Sa = Sa*_*T*_ *- Sa*_*0*_ .

### Fluoride release evaluation

For the fluoride release analysis, duplicate measurements were performed in 1 mL of erosive solution, with 1 mL of Total Ionic Strength Adjustment Buffer II (TISAB II) for each specimen. A fluoride-specific electrode (Orion 9609 BNW, ThermoScientific), previously calibrated using a standard calibration curve, was used to measure the fluoride ion concentration in the erosive solution, after immersing the specimens for 24 hours in an incubator (37°C ± 1°C). The mV readings were converted to μgF/mm² using the formula (released amount* total solution volume) / sample area. All the evaluations described above were performed by a trained operator.

### Statistical analysis

The data were tabulated and analyzed using Jamovi 2.5.5 software.^18^ After descriptive analysis, the normality of the distribution of all variables was assessed using the Shapiro-Wilk test. For intragroup analysis of paired samples, the paired t-test was used for groups with normal distributions (p>0.05), and the Wilcoxon test was used for those with non-normal distributions (p<0.05). The correlation between tests was evaluated using Pearson (rho) and Spearman (r) matrices, in the presence or absence of normality, respectively. For intergroup analyses, the data were subjected to the Kruskal-Wallis test and one-way ANOVA, with Tukey's Post Hoc test. The significance level was set at 5%.

## Results

*Measurement and calculation of initial thickness (d*_*0*_ *), final thickness (d*_*t*_ *), and eroded depth (d)*

In the intragroup analysis, all GICs showed a statistically significant difference between *D*_*0*_ and *D*_*T*_ values (p<0.05). For the eroded depth (*D*) values, VM showed the lowest (0.10 ± 0.03) and VD the highest (0.63 ± 0.07). In the intergroup analysis for *D*, all materials showed statistically significant differences among themselves (p<0.05). Considering the imported GIC GI, the eroded depth values (0.19 ± 0.01) were lower than those of MA and VD, but higher than those of VM (p<0.05). Only one GIC (VM) met the requirement of an eroded depth below 0.17 mm, as recommended by ISO 9917:2017 (Table 2).

**Table 2:** Eroded depth (*D*) results, in millimeters, of GICs after the erosive test.

GIC	*D*_0_	*D*_T_	*D*
Vidrion R	2.28 ± 0.06^Aa^	1.65 ± 0.11 ^Ba^	0.63 ± 0.07^w^
Maxxion R	2.21 ± 0.09^Ab^	1.89 ± 0.12 ^Bb^	0.32 ± 0.04^x^
Vitro Molar	2.11 ± 0.07^Ac^	2.01 ± 0.04 ^Bc^	0.10 ± 0.03^y^
GlasIonomer	2.24 ± 0.08^Ab^	2.04 ± 0.08 ^Bc^	0.19 ± 0.01^z^

*Different uppercase letters indicate an intragroup statistically significant difference (p<0.05). Different lowercase letters indicate a statistically significant intergroup difference (p<0.05). *D*_*0*_ - initial thickness; *D*_*T*_ - final thickness; *D* - eroded depth.

### *Measurement and calculation of initial mass (m*_*0*_ *), final mass (m*_*t*_ *), and eroded mass (m)*

Considering the mass value, in the intragroup analysis, all GICs showed a statistically significant difference between *M*_*0*_ and *M*_*T*_ values (p<0.05). For the eroded mass values (*M = M*_*T*_ *- M*_*0*_ ), VM showed the smallest mass loss (0.0113 ± 0.0037), whereas VD showed the largest (0.0428 ± 0.0044). In the intergroup analysis, GI showed a mass loss similar to that of MA (p>0.05) and different from VM and VD (p<0.05) (Table 3).

**Table 3:** Eroded mass (M) results, in grams, of GICs after the erosive test.

GIC	*M*_0_	*M*_T_	*M*
Vidrion R	0.0768 ± 0.0025^Aa^	0.0340 ± 0.0042^Ba^	0.0428 ± 0.0044^x^
Maxxion R	0.0742 ± 0.0043^Aa^	0.0470 ± 0.0044^Bb^	0.0272 ± 0.0039^y^
Vitro Molar	0.0740 ± .,0041^Aa^	0.0627 ± 0.0012^Bc^	0.0113 ± 0.0037^z^
GlasIonomer	0.0868 ± 0.0044^Ab^	0.0595 ± 0.0030^Bc^	0.0273 ± 0.0025^y^

*Different uppercase letters indicate an intragroup statistically significant difference (p<0.05). Different lowercase letters indicate a statistically significant intergroup difference (p<0.05). *M*_*0*_ - initial mass; *M*_*T*_ - final mass; *M* - eroded mass.

### Surface topography

**Figure 1:**
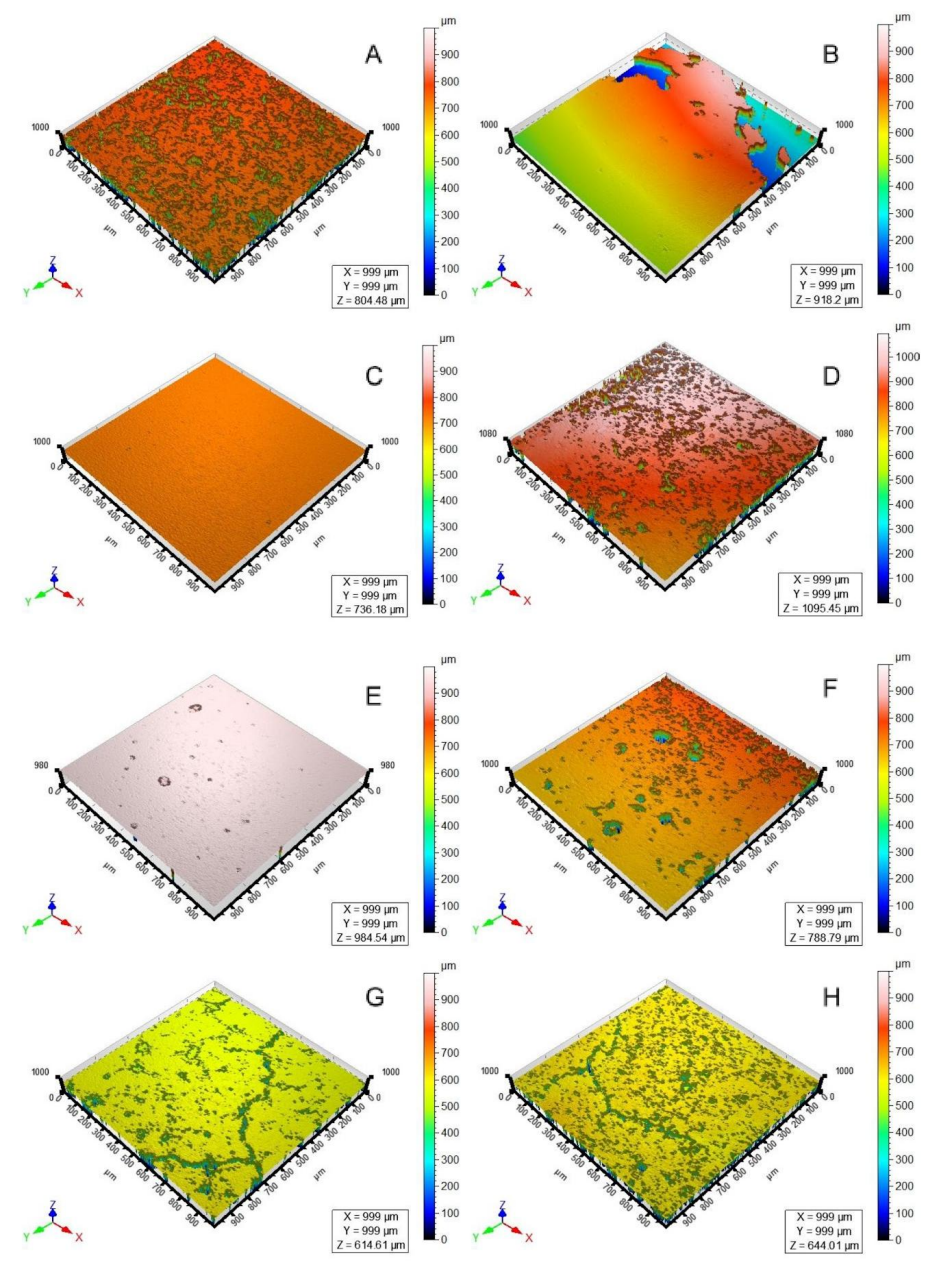
Surface topography images before and after the erosive challenge. A and B - VD images before and after the erosive challenge; C and D - MX images before and after the erosive challenge; E and F - VM images before and after the erosive challenge; G and H - GI images before and after the erosive challenge. VD - Vidrion R; MX - Maxxion R; VM - Vitro Molar; GI - GlasIonomer FX ULTRA.

Considering the linear roughness (*Ra*), in the intragroup analysis, only VD did not show a statistically significant difference between *Ra*_*0*_ and *Ra*_*T*_ values (p>0.05), showing a reduction in linear roughness. For the difference in linear roughness values (*Ra = Ra*_*T*_ *- Ra*_*0*_ ), MA, VM, and GI showed a significant increase in linear roughness after the erosive test (p<0.05) (Figure 1). In the intergroup analysis, MA showed the greatest variation in linear roughness compared to the other GICs (p<0.05). In contrast, GI and VM showed a similar increase in linear roughness (p>0.05) (Table 4). 

In relation to the volumetric roughness (*Sa*), in the intragroup analysis, VD and GI did not show a statistically significant difference between *Sa*_*0*_ and *Sa*_*T*_ values (p>0.05). For the difference in volumetric roughness values (*Sa = Sa*_*T*_ *- Sa*_*0*_ ), only MA and VM showed a significant increase in volumetric roughness after the erosive test, with MA showing the greatest variation (p<0.05). In the intergroup analysis, there was no statistically significant difference in volumetric roughness between groups (p>0.05) (Table 5). Comparative illustrative images of *Sa*_*0*_ and *Sa*_*T*_ for the groups are shown in Figure 1.

**Table 4:** Linear roughness (*Ra*) results, in micrometers, of GICs after the erosive test.

GIC	*Ra*_*0*_	*Ra*_*T*_	*Ra*
Vidrion R	40.96 ± 37.84^Aa^	23.00 ± 31.56^Aa^	17.96 ± 43.80^x^
Maxxion R	1.83 ± 1.84^Ab^	51.59 ± 33.10^Bbc^	- 49.76 ± 33.31^y^
Vitro Molar	5.62 ± 7.86^Ab^	24.88 ± 22.12^Bac^	-19.25 ± 22.87^x^
GlasIonomer	27.50 ± 22.20^Aa^	46.89 ± 18.75^Bb^	-14.53 ± 35.27^x^

*Different uppercase letters indicate an intragroup statistically significant difference (p<0.05). Different lowercase letters indicate a statistically significant intergroup difference (p<0.05). *Ra*_*0*_ - initial linear surface roughness; *Ra*_*T*_ - final linear surface roughness; *Ra* - eroded linear surface roughness.

**Table 5:** Volumetric roughness (*Sa*) results, in micrometers, of GICs after the erosive test.

GIC	*Sa*_*0*_	*Sa*_*T*_	*Sa*
Vidrion R	142.82 ± 127.82^Aa^	187.01 ± 179.12^Aa^	-44.19 ± 183.84^x^
Maxxion R	4.98 ± 2.69^Aa^	217.80 ± 73.16^Ba^	-212.82 ± 75.27^x^
Vitro Molar	38.29 ± 32.56^Aa^	154.56 ± 75.42^Ba^	-116.27 ± 65.11^x^
GlasIonomer	103.96 ± 81.74^Aa^	213.31 ± 88.77^Aa^	-109.35 ± 139.57^x^

*Different uppercase letters indicate an intragroup statistically significant difference (p<0.05). Different lowercase letters indicate a statistically significant intergroup difference (p<0.05). *Sa*_*0*_ - initial volumetric surface roughness; *Sa*_*T*_ - final volumetric surface roughness; *Sa* - eroded volumetric surface roughness.

### Fluoride release

Considering the fluoride release, MA showed the highest value (98.6 ± 11.8), while VM showed the lowest (38.4 ± 9.97). In the intergroup analysis, VD and MA showed similar fluoride release (p>0.05). GI showed a fluoride release value higher than that of VM (Table 6).

**Table 6:** Fluoride release results, in micrometers, of GICs after the erosive test.

GIC	Mean ± standard deviation
Vidrion R	91.4 ± 8.16^a^
Maxxion R	98.6 ± 11.8^a^
GlasIonomer	66.1 ± 4.13^b^
Vitro Molar	38.4 ± 9.97^c^

*Different letters indicate statistically significant difference (p<0.05).

### Investigation of the existence of correlation between variables

Considering the studied variables (thickness, mass, linear roughness, volumetric roughness, and fluoride release), no statistically significant differences were found, except for the relation between the fluoride release and volumetric roughness variables of GlasIonomer FX ULTRA® (strong and positive correlation) (tables 7 to 8 , 9 , 10).

**Table 7:** Correlation results between the variables for Vidrion R®.

		*M*	*D*	*Ra*	*Sa*	*Fr*
*M*	Spearman`s rho; p value	- -				
*D*	Spearman's rho; p value	0.086; 0.919	- -			
*Ra*	Spearman's rho; p value	- 0.257; 0.658	0.421; 0.082	- -		
*Sa*	Spearman's rho; p value	- 0.257; 0.658	- 0.486; 0.356	0.200; 0.714	- -	
*Fr*	Spearman's rho ; p value	- 0.657; 0.175	- 0.086; 0.919	0.029; 1.000	- 0.429; 0.419	- -

*Fr* - Fluoride release.

**Table 8:** Correlation results between the variables for Maxxion R^®^.

		*M*	*D*	*Ra*	*Sa*	*Fr*
*M*	Spearman`s rho; p value	- -				
*D*	Spearman's rho; p value	0.700; 0.233	- -			
*Ra*	Spearman's rho; p value	- 0.200; 0.783	0.136; 0.589	- -		
*Sa*	Spearman's rho; p value	0.700; 0.233	- 0.086; 0.919	- 0.657; 0.175	- -	
*Fr*	Spearman's rho; p value	0.900; 0.083	0.771; 0.103	0.486; 0.356	0.314; 0.564	- -

*Fr* - Fluoride release.

**Table 9:** Correlation results between the variables for Vitro Molar^®^.

		*M*	*D*	*Ra*	*Sa*	*Lf*
*M*	Spearman`s rho; p value	- -				
*D*	Spearman's rho; p value	0.278; 0.594	- -			
*Ra*	Spearman's rho; p value	- 0.031; 0.954	-0.230; 0.358	- -		
*Sa*	Spearman's rho; p value	- 0.772; 0.072	0.143; 0.803	0.143; 0.803	- -	
*Fr*	Spearman's rho; p value	0.772; 0.072	0.314; 0.564	- 0.371; 0.497	- 0.543; 0.297	- -

*Fr* - Fluoride release.

**Table 10:** Correlation results between the variables for GlasIonomer FX ULTRA^®^.

		*M*	*D*	*Ra*	*Sa*	*Fr*
*M*	Pearson's r; p value	- -				
*D*	Pearson's r; p value	- 0.235; 0.654	- -			
*Ra*	Pearson's r; p value	- 0.597; 0.211	0.165; 0.512	- -		
*Sa*	Pearson's r; p value	0.744; 0.090	- 0.676; 0.141	- 0.674; 0.142	- -	
*Fr*	Pearson's r; Valor de p	0.748; 0.087	- 0.620; 0.189	- 0.774; 0.071	0.832; 0.040*	- -

* p < .05. *Fr* - Fluorider elease.

## Discussion

In the present study, the null hypothesis was partially rejected because differences were observed among the properties of the studied materials; however, only the relationship between fluoride release and the volumetric roughness variables of GlasIonomer FX ULTRA showed a statistically significant difference. 

According to ISO 9917:2007^19^, the limit value for the eroded depth of a glass ionomer cement subjected to erosive testing is no more than 0.17 mm. In this study, only one GIC (VM) showed an eroded depth below this value, reaching adequate erosive resistance defined by ISO. This raises concerns about the suitability of the other three products for use in clinical practice. In the studies by Bueno et al.^12^ and Navarro et al.^10^, Maxxion R also showed the highest eroded depth values, but it was the only one to exceed the ISO-defined values. 

To formulate standardized technical protocols of international scope across various fields of knowledge, the International Organization for Standardization (ISO) was established in 1947. This entity issues standards that enable test reproducibility and product quality.^20^ Although neither the ISO nor expert consensus^12^ defined them as essential, the present study employed mass loss measurements and surface topography assessments-specifically linear and volumetric roughness-as complementary methods to better understand the effects of erosive challenges on GICs. Once again, the national material Vitro Molar exhibited the best performance in terms of eroded mass. The absolute values of eroded mass followed the same trend as the absolute values of eroded depth.

Regarding surface topography, linear roughness was significantly higher for Maxxion R than for the others. It is interesting to note that this GIC had the lowest initial absolute values and excellent surface smoothness; however, it showed the highest final absolute values, indicating greater erosive loss, which could negatively impact the longevity of the restoration. Another important point is that Vidrion R did not differ statistically from other GICs but showed the opposite behavior, with a final absolute value lower than the initial absolute value. Since this material had very high initial roughness values and significant erosive loss, it is suggested that significant particle detachment occurred during the erosive challenge, helping reduce its roughness and thus biasing the results, thereby not revealing the actual structural loss. This finding demonstrates the importance of considering the surface's initial conditions and erosive loss to avoid misinterpreting the results. 

Regarding volumetric roughness, although the materials did not differ statistically, Maxxion R again showed the greatest variation, with excellent initial absolute values but extremely high final absolute values. Although the imported GlasIonomer FX ULTRA and the national Vidrion R showed increases in volumetric roughness, no statistically significant difference was observed between their initial and final measurements. This outcome is likely due to their already high initial roughness, which may predispose restorations to staining and biofilm accumulation in clinical situations. 

The topographic analysis was qualitative, and, given that glass ionomer cement is a porous material, it was assessed before and after the erosive challenge to minimize bias. 

In the oral environment, restorative materials are constantly exposed to various acids, whether of extrinsic or intrinsic origin. The more acidic the environment, the greater the fluoride release from the material^12^, ^17^, ^21^ During the remineralization process, available fluoride can be incorporated into the dental structure, forming fluorapatite, which is less soluble than hydroxyapatite ^21^, ^22^, ^23^, thereby helping reduce demineralization. However, in acidic environments, surface degradation of restorative materials intensifies, leading to increased surface roughness. This, in turn, may contribute to restoration staining, microbial colonization, and plaque accumulation, potentially leading to gingival inflammation and the onset of oral diseases.^15^ ^,^ ^16^ Therefore, achieving an optimal balance between acid erosion resistance and fluoride release is essential.

In terms of fluoride release, this study found that the national GICs Vidrion R and Maxxion R showed the highest values, releasing large amounts, while the imported GlasIonomer FX ULTRA had an intermediate value, and the national Vitro Molar presented the lowest values. Although no correlation was found between eroded mass and fluoride release, it is interesting to note that the GICs with the highest and lowest absolute values of eroded mass also showed the lowest and highest absolute values of fluoride release, respectively. Bueno et al.^12^ and Navarro et al.^10^ obtained results showing the highest fluoride release values for Maxxion R and the lowest for Vidrion R. It is suggested that methodological differences in measuring fluoride release between the previously cited studies and the present investigation may have contributed to the discrepancies in the results. The absence of standardized protocols for fluoride release testing and reporting makes it difficult to make direct comparisons across studies.^12^ ^,^ ^24^ Consequently, experts have established the release of the highest possible amount of fluoride as a desirable objective.^10^

Previous studies emphasized that the powder/liquid ratio affects acid erosion and fluoride release, with higher ratios associated with lower acid erosion and fluoride release.^10^ ^,^ ^12^ In the present study, the only exception to this rule was Vidrion R, which, although it had a powder/liquid ratio that classified it as a high-viscosity GIC (>3.6), showed greater acid erosion and the highest fluoride release values. Despite that, Bueno et al.^12^ considered that the amount of fluoride released by a material and the ease of diffusion are determined by factors such as the reaction kinetics and its composition. Thus, it is emphasized that materials with high fluoride content may not release fluoride in large initial amounts, as the fluoride may be trapped within the matrix and released more slowly. They also state that a reduced P/L ratio increases the amount of water in the matrix, resulting in mechanically weaker cements, greater susceptibility to acid erosion, and greater permeability to diffusing fluoride ions. On the other hand, Pardi et al.^25^ highlight that fluoride release by a material is determined by its matrix, its setting mechanism, and the amount of fluoride present in its composition. In this perspective, we suggest that the chemical differences between the materials and the short-term evaluation of their fluoride release (24h after immersion in an erosive solution) explain the results.

In glass ionomer cement, fluoride is a component of the glass, which is a mixture containing calcite (CaF_2_) or cryolite (Na_3_AlF_6_). The fluoride present in the composition may improve the appearance of the cement after setting by reducing its refractive index and may also enhance compressive strength.^17^ ^,^ ^21^ Despite this, Bueno et al.^12^ highlight that fluoride destabilizes the glass network, contributing to the partial loss of fluoride directly from the glass. Tuygunov et al. ^26^ state that sustained release of fluoride can be primarily attributed to the gradual dissolution of unreacted or partially reacted glass particles entrapped within the matrix. Therefore, future research is needed to determine whether there is a significant relationship between the percentage of glass in the composition of the materials studied and the amount of fluoride released.

Studies also reported a positive correlation between acid erosion and fluoride release ^10^, ^12^, which was not observed in the present study. Once again, the divergence in methodology for measuring fluoride release is suggested as a possible cause for this difference in results. However, as in the two previous studies mentioned, it is considered desirable for the material to achieve the highest possible fluoride release, provided it does not cause significant loss due to acid erosion. Thus, although the national GIC Vitro Molar showed the lowest fluoride release, it performed well, releasing reasonable amounts of fluoride without compromising its integrity, as evidenced by its lowest mass loss and by being within the ISO-defined limit for acid erosion.

Although widely used and possessing beneficial characteristics, GICs also present disadvantages, such as lower microhardness and lower resistance to tensile stress and wear. ^27^, ^28^, ^29^ These mechanical properties are essential for restorative materials, especially in posterior tooth cavities. In an attempt to improve the mechanical performance of these materials, various strategies have been developed in recent years, such as the incorporation of other components into the formula ^22^, ^30^, changes in the powder/liquid ratio^31^ ^,^ ^32^, particle size^33^, and polyacid molecular weight(26). Changes in the powder-to-liquid ratio alter the material's viscosity and mechanical properties.^31^ ^,^ ^32^ ^,^ ^34^ Studies have shown that the presence of smaller particles improves wear resistance, material hardness, and compressive strength by allowing greater homogeneity of the matrix.^31^ ^,^ ^33^ Tuygunov et al.^26^ demonstrated that reducing the particle size of GIC powders significantly enhanced fluoride release, which can be attributed to an increase in reactive surface area, exposing the surfaces to the aqueous environment. In terms of polyacid molecular weight, studies have shown that longer polyacid chains result in lower erosion rates, greater solubility, and reduced material degradation, as they form a denser, cross-linked structure that offers resilience against acid penetration.^35^ ^,^ ^36^ However, the amounts of each component in the cements, as well as the exact particle sizes and polyacid molecular weights used, are not known, as their formulas are proprietary. Further research considering the cited strategies and the studied materials should help to improve their performance regarding the tests assessed. 

It is important to note that the GlasIonomer used in the expert consensus test^10^ and in the study by Bueno et al.^12^ was FX-II, whereas in this work, it was FX ULTRA. According to the manufacturer of GlasIonomer FX ULTRA, this material is a more current and improved version of GlasIonomer FX-II. Its advantages over other products in the same category should include advanced aesthetics due to greater translucency, being indicated for Class III and V restorations, as well as resistance (evaluated in compressive testing) and durability, with indications for intermediate posterior restorations in Class I and II. They also state that greater translucency, color stability, and tone mimicry are ensured by the incorporation of a special glass in its formula.^37^ ^,^ ^38^ However, this CIV has a considerably higher cost than the national CIVs, and more studies are needed to compare other properties and assess its cost-effectiveness.

The limitations of this study include the lack of methodological standardization for some of the evaluated parameters, such as fluoride release, and the absence in the scientific literature of studies addressing the properties and characteristics of the GlasIonomer FX ULTRA, which contributed to the difficulty of comparing with previous studies. Also, fluoride release was assessed only at 24h, which limits the interpretation and comparability. Furthermore, although significant results with clinical relevance were found, as this is an *in vitro* study, it does not perfectly reproduce the conditions of the oral environment, and future clinical studies are needed.

## Conclusion

Therefore, considering all the analyses in terms of clinical relevance, the national material Vitro Molar showed the best performance, as it releases a reasonable amount of fluoride without significant structural loss, which prevents the infiltration and accumulation of microorganisms and allows the material to have a longer lifespan in the oral cavity.

## Data Availability

The research data are available upon request.
